# ^1^H-NMR Metabolomics and LC-MS Analysis to Determine Seasonal Variation in a Cosmeceutical Plant *Leucosidea sericea*

**DOI:** 10.3389/fphar.2020.00219

**Published:** 2020-03-05

**Authors:** Phophi Freda Sehlakgwe, Namrita Lall, Gerhard Prinsloo

**Affiliations:** ^1^Department of Agriculture and Animal Health, University of South Africa, Johannesburg, South Africa; ^2^Department of Plants and Soil Sciences, Plant Science Complex, University of Pretoria, Pretoria, South Africa; ^3^School of Natural Resources, University of Missouri, Columbia, MO, United States

**Keywords:** metabolomics, *Propionibacterium acnes*, seasonal, flavonoid, polymethoxy flavone, anti-bacterial, anti-acne, *Leucosidea sericea*

## Abstract

*Leucosidea sericea* is an evergreen shrub belonging to the Rosaceae family with previous studies that indicated that *L. sericea* extracts exhibited strong anti-bacterial properties against *Propionibacterium acnes*, showing potential as a cosmeceutical. The plant is traditionally used as a vermifuge, as an astringent and to treat conjunctivitis. Commercial production is, however, not possible as no information is available on cultivation and the effect of external environmental factors such as seasonal variation on the medicinal properties of the plant. Seasonal variation was investigated and it was found that significant differences were observed between the anti-acne (*P. acnes*) activity of plant material collected in different seasons. The best activity was found in winter with a mean minimum inhibitory concentration (MIC) of 5.20 μg mL^–1^ compared to spring at 26.04 μg mL^–1^. A ^1^H NMR-based untargeted metabolomic analysis was used to determine the differences in the chemical profiles of plant samples collected in different seasons. Principal component analysis (PCA) showed clear separation of the seasons and a supervised orthogonal partial least square discriminant analysis (OPLS-DA) was used to determine the compounds that differentiated the spring from the winter samples. The contribution plot indicated a strong positive association with the NMR regions from δ 1.2–1.6, 3.3–4.1, and 6.8–8.0 ppm indicative of a compound with an aromatic ring. Different LC-MS analyses were used in conjunction with a compound database, MAGMa and CSIFingerID, which led to the identification of the compound 2-(4-ethoxyphenyl)-5,6,7,8-tetramethoxy-4H-1-benzopyran-4-one and also confirmed the presence of tangeritin, rutin, quercetin glucoside, and kaempferol glucosides as well as several other compounds previously identified from the plant. This compound similar in structure to the anti-microbial flavonoid tangeritin, was only present in the winter samples. It is therefore recommended that seasonal variation be closely monitored during cultivation and commercial harvesting, and that winter is the preferred harvesting season to obtain the best anti-acne activity.

## Introduction

*Leucosidea sericea* Eckl. & Zeyh. is an evergreen dense shrub belonging to the family Rosaceae which includes the genus Rosa, poorly presented in South Africa with *L. sericea* the sole representative of the genus *Leucosidea*. The tree also occurs in mountainous areas near water at high altitudes of 1000 m in parts of Africa such as Zimbabwe and Lesotho. It is commonly known as “oldwood” (English), “ouhout” (Afrikaans), and “umTshitshi” in Zulu, and traditionally used as a charm to protect the inhabitants of homesteads, treat various ailments such as opthalmia and intestinal worm infection, used as a vermifuge and astringent. The antioxidant, anti-inflammatory, acetylcholinesterase inhibitory activity and mutagenic activities have been investigated in different studies, although the cosmeceutical application is poorly researched with a single report on the anti-acne activity (Aremu et al., 2010; Sharma et al., 2014).

Acne is a common skin disease, affecting adolescent males and females, although it can present even in their twenties (Sinha et al., 2014). Topical retinoids, topical antimicrobials, isotretinoin and oral antibiotics are the most commonly used methods of treating acne, but even though there is a wide range of acne treatments, the response of acne to treatment varies considerably (Kim et al., 2008b). Additionally, bacterial resistance is a continuous challenge and plants therefore provide various opportunities to find new anti-acne agents (Chomnawang et al., 2007; Tsai et al., 2010; Sinha et al., 2014). The anti-acne activity for *L. sericea* was reported with a MIC value of 15.62 μg mL^–1^ and the isolated active compound α-kosin with an MIC value of 1.95 μg mL^–1^ (Sharma et al., 2014), and therefore presents and opportunity as an anti-acne cosmeceutical.

Metabolomics is used in a wide range of applications such as plant breeding, assessment of crop quality, food assessment, toxicity assessment, nutrition assessment, medical diagnosis, assessment of disease status, pharmaceutical drug developments, gene-function elucidation, integrated systems biology, and technological advances in analytical chemistry (Moco et al., 2007). Untargeted metabolomics, provides a unique tool to investigate relatively unknown plants, to determine the effects of external stimuli such as season on the chemical profile, and associated biological activity. Since abiotic factors such as ultra violet light, temperature and biotic factors such as parasitism and pathogenic attack influence the development of complex metabolic compounds in plants, untargeted analysis ensure that all changes potentially be observed and included in quality assessment parameters (Hardy and Hall, 2012). A combination of different analytical techniques such as NMR and LC-MS provides support for the identification of changes in chemical profile and for the identification of compounds responsible for the altered biological activity.

Although *L. sericea* is used against various ailments, the phytochemical research on this plant is very limited and an untargeted NMR-based metabolomics and LC-MS analysis were used to investigate metabolite variation amongst seasons (winter, summer, spring, and autumn) in *L. sericea* leaves and how it affected the biological activity. LC-MS analyses were used to identify the annotated compounds from the untargeted metabolomic analysis; as well as several known compounds and compounds not identified in the plant before. The changes in chemical profile and associated biological activity is proposed to be linked to an increase in concentration of a polymethoxy flavonol compound in winter, indicating the need for systematic research to determine optimum conditions for harvesting of material to obtain the best biological activity.

## Materials and Methods

### Collection of Plant Material

Leaves of *L. sericea* were collected in four consecutive seasons namely; October 2013 (spring), January 2014 (summer), April 2014 (autumn) and June 2014 (winter) from uniform, healthy vigorous plants from the Clearwater Florida region, South Africa (26°9′26″S 27°54′10″E) and compared to the voucher specimen PRU (119052) deposited at H. G. W. J. Schweickerdt Herbarium, Department of Plant Science, University of Pretoria, Pretoria. The collected leaves were then air-dried at room temperature, protected from direct sunlight. The dried leaves were ground into a fine powder and stored at room temperature in airtight containers protected from direct sunlight until analyses.

### Anti-acne Assay

The powdered plant material (3 g) were soaked in 30 mL of ethanol for 72 h and placed on a shaker at room temperature for extraction of the compounds for the anti-bacterial assay. The extracts were filtered and the filtrates were placed under laminar flow to evaporate the remaining solvent to produce around 10 mg of crude ethanol extract. The extract stock solution was prepared by weighing 2 mg of the ethanolic plant extract in a 2 mL Eppendorf tube. One mL of 10% DMSO (dimethyl sulfoxide- C_2_H_6_OS) was added and sonicated at 40°C for 5 min, thereafter addition of 100 μl of double distilled water (ddH_2_O). Minimum inhibitory concentration (MIC) values of ethanolic extracts of *L. sericea* samples were determined for anti-bacterial activity against *Propionibacterium acnes* using a serial dilution method (Eloff, 1998). Bacterial cultures of *P. acnes* were grown on Brain Heart Infusion agar [Merck SA (Pty) Ltd.] and transferred to Nutrient Broth [Merck SA (Pty) Ltd.] using a sterile inoculation loop. The OD (optical density) of the bacterial suspension was then adjusted to 0.132 at 600 nm using a Beckmann spectrophotometer, corresponding to 0.5 McFarland standard equating to 108 CFU mL^–1^. Tetracycline was used as a positive control (Sigma-Aldrich- Kempton Park, South Africa) prepared by adding 2 mg in 10 ml autoclaved dH_2_O. A volume of 100 μL was then added in a serial dilution method to give concentrations of 500–3.96 μg mL^–1^ of the plant extracts and positive control wells in the sterilized 96-well plate. A bacterial suspension was included as a negative control and DMSO was used as a solvent control to make sure that bacterial inhibition was due to the sample activity and not the solvent vehicle.

The 96-well plates were incubated for 72 h at 37°C under anaerobic conditions in Anaerocult A [Merck SA (Pty) Ltd.] in the dark. Twenty μL of PrestoBlue was added to determine the MIC values by visually observing the color change in the wells. Since PrestoBlue is a resazurin-based dye blue in color, it converts to resorufin (pink) in the presence of viable bacterial cells. The MIC values of the samples were defined as the lowest concentration of the plant extract that inhibits the conversion of PrestoBlue from blue to pink.

### Metabolomic Analysis

The same dried and grounded material used for the anti-acne assay was used for the NMR-based metabolomic analysis. An untargeted metabolomics analysis method was adapted (Maree and Viljoen, 2012; Mediani et al., 2012) and the plant material was subsequently extracted using a direct extraction method, where ethanol was replaced with methanol:water. Fifty milligrams of the dried leaves were weighed in 2 mL Eppendorf tubes and then extracted with 0.75 mL of deuterated methanol (CH_3_OH-d4) and 0.75 mL of potassium dihydrogen phosphate (KH_2_PO_4_) buffer in deuterated water (D_2_O) containing 0.1% (w/w) TSP (trimethylsilylpropionic acid sodium salt). The desired pH (6.0) was obtained adding NaOD (deuterated sodium hydroxide) to the buffer solution. The samples were then vortexed for 1–2 min at room temperature, sonicated at 30°C for 20 min and then centrifuged at 13 000 rpm for 20 min (Kim et al., 2010). The supernatant from each tube was then transferred into 5 mm NMR tubes where 32 scans were performed on a 600 MHz NMR spectrometer (Varian Inc., California, CA, United States).

The spectral data from NMR was processed using MestReNova software (9.0.1, Mestrelab Research Spain) where the spectral data from NMR was subjected to phase correction, baseline correction, referencing and normalizing after which the spectral intensities were reduced to integrated regions (bins) of equal width (0.04 ppm each) corresponding to the region of 0.04–10.00 ppm. The water peak (4.70–4.90 ppm) and methanol peaks (3.30–3.36 ppm) were excluded from the final data for further analysis (Mediani et al., 2012). The data from MestReNova was exported to Microsoft Excel for multivariate data analysis using SIMCA-P software (13.0, Umetrics, Sweden). The data was first analyzed using an unsupervised method known as principal component analysis (PCA), followed by a supervised model, and orthogonal partial least square discriminatory analysis (OPLS-DA). A scores plot and a contribution plot were used to determine sources of variation and the NMR values from the plots were then used in conjunction with databases and published literature for annotation. The permutation test with 100 permutations was performed for validation of the OPLS-DA model.

### Liquid Chromatography-Mass Spectroscopy (LC-MS)

Liquid Chromatography-Mass Spectroscopy was used in order to confirm the proposed compound annotated from NMR-metabolomics and chemometric studies as well as to confirm the presence of compounds previously identified in the plant. The dried leaf material was ground and 1 g was extracted with 1.5 ml MeOH (analytical grade, Sigma-Aldrich-Kempton Park, South Africa). Samples were sonicated in an ultrasonic bath for 15 min and centrifuged for 15 min at 15 000 rpm. The supernatant was filtered through 0.2 micron syringe filters (Sartorius Minisart RC 4). Extractions were performed in triplicate for statistical processing.

#### UHPLC Analysis

A Waters UHPLC coupled in tandem to a Waters SYNAPT G1 HDMS mass spectrometer was used to generate accurate mass data. Optimization of the chromatographic separation was done utilizing a Waters HSS T3 C18 column (150 mm × 2.1 mm, 1.8 μm) and the column temperature controlled at 60°C. A binary solvent mixture was used consisting of water (Eluent A) containing 10 mM formic acid (natural pH of 2.3) and acetonitrile (Eluent B) containing 10 mM formic acid. The initial conditions were 90% A at a flow rate of 0.4 mL min^–1^ and were maintained for 1 min, followed by a linear gradient to 1% A at 35 min. The conditions were kept constant for 2 min and then changed to the initial conditions. The runtime was 40 min and the injection volume was 1 μL. Samples were kept at 6°C in the Sample Manager during the analysis.

#### Quadrupole Time-of-Flight Mass Spectrometry (q-TOF-MS) Analyses

A SYNAPT G1 mass spectrometer was used in V-optics and operated in electrospray mode to enable detection of phenolic and other ESI-compatible compounds. Leucine enkephalin (50 pg mL^–1^) was used as reference calibrant to obtain typical mass accuracies between 1 and 5 mDalton (mDa). The mass spectrometer was operated in both ESI positive and negative modes with a capillary voltage of 2.5 kV, the sampling cone at 30 V and the extraction cone at 4.0 V. The scan time was 0.1 s covering the 50 to 1200 Dalton mass range. The source temperature was 120°C and the desolvation temperature was set at 450°C. Nitrogen gas was used as the nebulization gas at a flow rate of 550 L h^–1^ and cone gas was added at 50 L h^–1^. MassLynx 4.1 (SCN 872) software was used to control the hyphenated system and to perform all data manipulation.

### Compound Annotation and Identification

#### NMR Data

The important chemical shifts from the untargeted NMR-based metabolomic analysis, identified from the contribution plots, were compared to the chemical shifts of compounds in databases such as Chenomx (Version 8.3) and the Human Metabolome Database^1^. Candidate structures were compared with previously published literature.

#### LC-MS Data

Automated chemical structure annotation and identification was obtained using MAGMa^2^ to propose the most likely structure of the unknown compound based on the fragmentation pattern (Ridder et al., 2014, 2013). The fragmentation trees obtained from MAGMa were then transferred to CSIFingerID^3^ to confirm the identity based on the fragmentation pattern of the proposed compound. CSIFingerID calculates fragmentation trees from known reference spectra, fragmentation tree similarities as well as PubChem (CACTVS) and Klekota–Roth fingerprints.

The raw data was processed and extracted ion chromatograms (XICs) obtained based on the newly proposed unknown compound and known compounds found in published literature. The accurate mass data of each detected compound was submitted for elemental composition, double bond equivalence (DBE) as well as isotopic fit calculations. Compound identification was further enhanced by analyzing all samples with low and high collision energy settings of the collision cell. To minimize compound fragmentation a low energy setting of 3 eV was used, but to enhance fragmentation of molecules various collision energies between 10 and 40 eV were used (MSe). Fragmentation spectra were submitted to the NIST mass spectral library (Stein, 2014, Version 2.2 build Jun 2014) as well as the mass spectral libraries developed on the Synapt G1 system for identification of the compounds.

## Results

The anti-acne assay achieved inhibitory activity at 3.90–31.25 μg mL^–1^ which is comparable to previous reports by Sharma et al. (2014) at a MIC value of 15.62 μg mL^–1^. Better activity was, however, obtained in the colder seasons namely autumn and winter (Table 1).

**TABLE 1:** MIC values of *L. sericea* extract against *Propionibacterium acnes* with MIC values in μg mL-1 for the four seasons.

Season	Mean (μg mL^–1^)	Std. dev.
Spring	26.04	9.02398
Summer	20.83	9.02398
Autumn	7.81	0
Winter	5.20	2.25744

*Each value represents the average of three replicates.*

Chenomx database and previously published data were used to annotate the main compounds in the plant extract (Table 2).

**TABLE 2:** Annotated compounds in the methanolic water extracts analyzed using a 600 MHz NMR.

Compound	NMR values of extract (ppm)	Chenomx database (ppm)
Alanine	1.46	1.46
	1.47	1.47
Acetamide	2.00	2.00
	nd	6.79
	nd	7.52
Choline	3.19	3.19
	nd	3.50–3.52
	nd	4.04–4.07
Glycine	3.54	3.54
Fumarate	6.52	6.52
Isoleucine	0.99	0.99
	1.01	1.01
	nd	1.22–1.28
	nd	1.42–1.48
	nd	1.96–1.98
	nd	3.66
Sucrose	3,44	3.46
	nd	3.54–3.57
	3.64	3.67
	3.71–3.88	3.74–3.90
	4.01	4.04
	4.14	4.20
	4.16	4.22
	5.39	5.40
	5.40	5.41
Xylose	3.22	3.22
	3.42	3.42
	nd	3.60–3.69
	nd	3.90–3.94
	4.56	4.56
	4.58	4.58
	5.17	5.17
	5.18	5.18

*When peaks of the extract were too small to match to the peaks of the pure compound, they were denoted as not detected (nd).*

**FIGURE 1:**
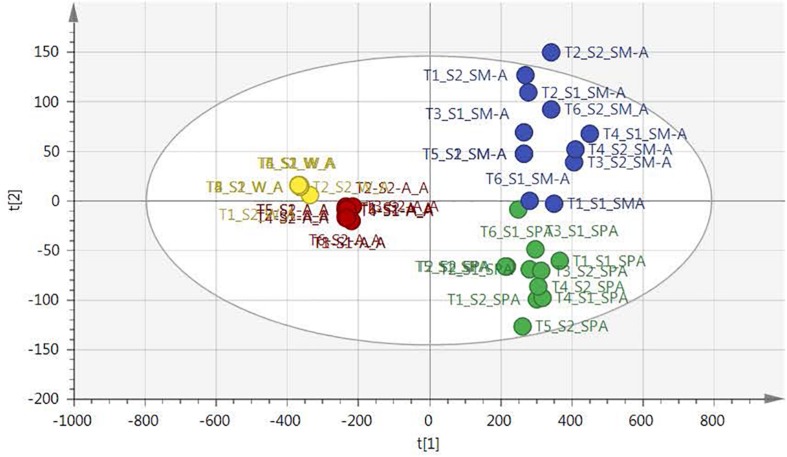
PCA scores plot of *Leucosidea sericea* leaves classified by seasons: summer (blue), spring (green), autumn (red), and winter (yellow).

NMR-based metabolomic analysis was used to determine the extent of the chemical changes in the plants that affected the anti-acne activity as was obtained in Table 1. The unsupervised PCA revealed clustering of samples based on the season of collection. Autumn and winter samples grouped close to each other, separated from the summer and spring samples (Figure 1), indicating a similar chemical profile of the samples in winter and autumn when compared to the summer and spring samples. The tight clusters for the winter and autumn samples indicated minimal changes in the chemical profiles for the samples collected in these seasons. This is contrasted to the variation of the sample distribution for the spring and summer samples, which were not tightly clustered but spread out with a slight overlap between the samples, indicative of more variation in the chemical profiles of these samples. The very good description of the variation in the samples and the predictability of the model is depicted in the *R*^2^ and Q^2^ values, respectively (*R*^2^ = 0.977 and *Q*^2^ = 0.955).

**FIGURE 2:**
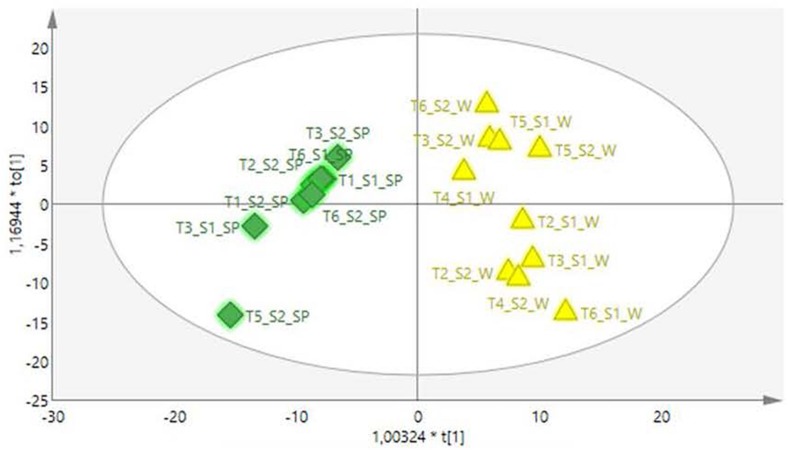
A OPLS-DA scores plot showing separation of the spring (green) and winter (yellow) samples.

**FIGURE 3:**
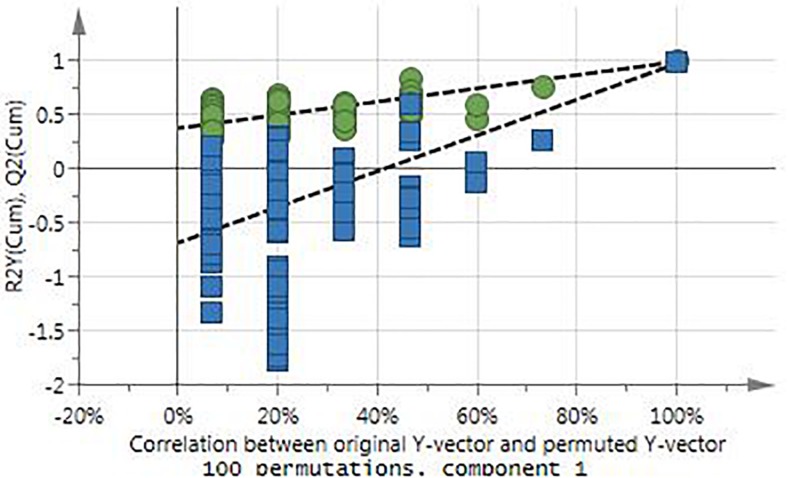
Validation of the OPLS-DA model with the permutation test.

Since the best and lowest activity was achieved in winter and spring seasons, respectively, a supervised analyses was conducted using only the spring and winter samples (Figure 2). The OPLS-DA score plot clearly separated the spring from the winter samples with a very good description of the variation and predictability score for the model (*R*^2^X = 0.914 and *R*^2^Y = 0.897). Due to the good separation of the samples in the OPLS-DA analysis, a contribution plot was constructed to determine the important NMR regions contributing to the separation of the samples into the two clusters. For validation of the model, the permutation test with 100 permutations showed that the model is valid (Figure 3).

**FIGURE 4:**
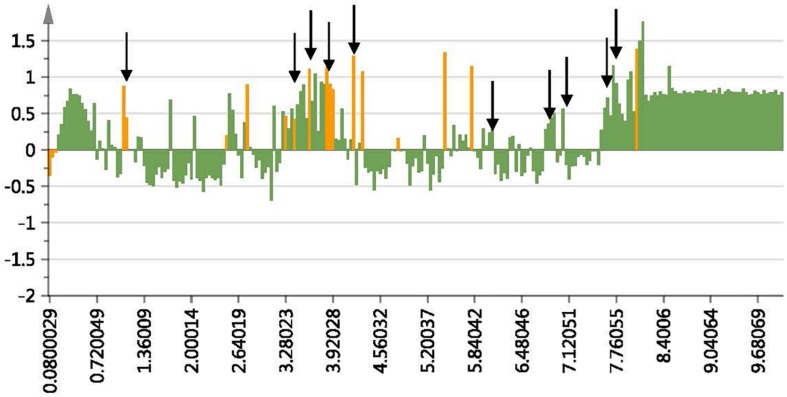
Contribution plot showing NMR regions responsible for the separation of the winter samples from the spring samples. The positive bars indicate the NMR regions contributing to the separation of the winter samples. The arrows indicate the NMR regions of the proposed compound 2-(4-ethoxyphenyl)-5,6,7,8-tetramethoxy-4H-1-benzopyran-4-one at δ 1.29, 3.66, 3.80, 4.08, 6.35, 6.8, 6.88, 7.67, and 7.71 ppm.

The contribution plot of the untargeted OPLS-DA analysis was used to locate the NMR regions of interest (Figure 4). The important ^1^H-NMR chemical shift regions were identified as δ 1.2–1.3, 3.2–4.1, 6.1–7.2, and 7.5–8.0. These regions are indicative of a flavonoid type structure with peaks in the aliphatic region (δ 1.2–1.3) and aromatic region (δ = 6.1–7.2 and 7.5–8.0) representative of a substituted aromatic ring.

**FIGURE 5:**
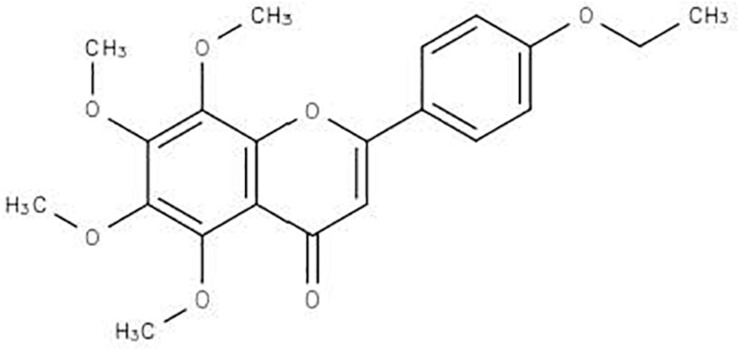
Structure of 2-(4-ethoxyphenyl)-5,6,7,8-tetramethoxy-4H-1-benzopyran-4-one.

After confirming the important NMR regions contributing to the clustering of the samples, and putative annotation of a chemical structure, LC-MS analysis was conducted to obtain more information on the proposed flavonoid type compound. A search for differences in the LC-MS profiles was conducted for the winter and spring samples, and possible peaks were identified based on the putative annotation obtained from the NMR-based metabolomic analysis. MAGMa was used to produce a hit list of possible structures that fit the NMR profile. Only one flavonoid type structure was present (from the peaks differentiating the samples of the different seasons), namely 2-(4-ethoxyphenyl)-5,6,7,8-tetramethoxy-4H-1-benzopyran-4-one (Figure 5) and was further investigated using CSIFinger ID. The fragmentation pattern and intensities were submitted to CSIFinger ID to determine the possible compounds that could be linked to the pattern and intensities. The same compound was proposed and the proposed compound’s NMR peaks were compared to the contribution plot as well as matched to the NMR profiles of the winter samples. The predicted NMR values of 2-(4-ethoxyphenyl)-5,6,7,8-tetramethoxy-4H-1-benzopyran-4-one^4^ (Banfi and Patiny, 2008), the NMR values found in the sample as well as the published NMR peaks of tangeretin (Rashed et al., 2015) are provided in Table 3. Additionally the correlation values of the correlation matrix functionality of SIMCA was used to determine the correlation values of the peaks of the proposed compound and is also presented in Table 3.

**TABLE 3:** NMR values of 2-(4-ethoxyphenyl)-5,6,7,8-tetramethoxy-4H-1 -benzopyran-4-one, the observed NMR values, the NMR values of tangeretin as well as the correlation values obtained from SIMCA.

H	Predicted (Banfi and Patiny, 2008)	Observed	Correlation values	Tangeretin (Rashed et al., 2015)
3	6.435 s	6.35 s	0.898	6.63 s
2′	7.533 ddd	7.67 d	0.789	7.85 d
3′	6.94 ddd	6.81 d	0.891	7.0 d
5′	6.94 ddd	6.88 d	0.897	7.0 d
6′	7.533 ddd	7.71 d	0.701	7.85 d
OCH_3_	3.831 s	3.81 s	0.831	4.07 s
OCH_3_	3.716 s	3.66 s	0.807	4.01 s
OCH_3_	3.838 s	3.67 s	0.833	3.92 s
OCH_3_	3.704 s	3.80 s	0.820	3.92 s
OCH_3_				3.86 s
OEth	4.177 q	4.08 m	0.857	
	1.277 t	1.29 t	0.855	

The LC-MS analysis further identified 15 compounds from this plant, based on comparison with a compound database (Table 4). The list of compounds includes compounds that were previously identified from the plant such as α-kosin, 3,5,7,3′,4′-pentahydroxyflavone and tiliroside, as well as compounds not previously identified from this plant including compounds such as tangeritin, rutin, quercetin glucoside, and kaempferol glucosides (Figure 7).

**TABLE 4:** Compounds in *Leucosidea sericea*, indicating the mode [ESI (–) and ESI (+)], formula, mass and the retention time.

	Compound	Formula	Mass	ESI (+)	ESI (–)	Mode and Rt (min)
1	3,5,7,3′,4′pentahydroxyflavone	C_15_H_10_O_7_	302.0427	303.0505	301.0348	ESI−; Rt 1.49
2	Procyanidin B1 and B2	C_30_H_26_O_12_	578.1424	579.1503	577.1346	ESI+/ESI−; Rt 2.36 and 3.50
3	Tangeritin	C_20_H_20_O_7_	372.1209	373.1287	371.1131	ESI−; Rt 6.45
4	5,7-dihydroxychromone	C_9_H_6_O_4_	178.0266	179.0344	177.0188	ESI−; Rt 8.55
5	Rutin	C_27_H_30_O_16_	610.1534	611.1612	609.1456	ESI+/ESI−; Rt 9.09
6	Quercetin glucoside	C_21_H_20_O_12_	464.0955	465.1033	463.0877	ESI+/ESI−; Rt 9.60
7	Kaempferol-3-O-rutinoside (Nicotiflorin)	C_27_H_30_O_15_	594.1585	595.1663	593.1507	ESI+/ESI−; Rt 11.81
8	Kaempferol-7-O-β-D-glucopyranoside	C_21_H_20_O_11_	448.1006	449.1084	447.0927	ESI+/ESI−; Rt 12.20
9	2-(4-ethoxyphenyl)-5,6,7,8-tetramethoxy-4H-1-benzopyran-4-one	C_21_H_22_O_7_	386.1366	387.1444	385.1287	ESI−; Rt 14.89
10	Tiliroside	C_30_H_26_O_13_	594.1373	595.1452	593.1295	ESI−; Rt 17.44 or 17.91
11	Desaspidinol	C_11_H_14_O_4_	210.0892	211.0970	209.0814	ESI−; Rt 18.95
12	Aspidinol B (Aspidinol)	C_12_H_16_O_4_	224.1049	225.1127	223.0970	ESI−; Rt 22.20
13	Aspidinol C or Aspidinol D	C_13_H_18_O_4_	238.1205	239.1283	237.1127	ESI−; Rt 24.15
14	1-hydroxy-2-oxopomolic acid	C_30_H_46_O_6_	502.3294	503.3373	501.3216	ESI−; Rt 24.77 or 26.97
15	α-kosin	C_25_H_32_O_8_	460.2097	461.2175	459.2019	ESI−; Rt 32.67

## Discussion

*Leucosidea sericea* was previously identified as a plant with good activity against *P. acnes* at 15.62 μg mL^–1^ and the isolated active compound α-kosin with an MIC value of 1.95 μg mL^–1^ (Sharma et al., 2014). Additionally 3,5,7,3′,4′-pentahydroxyflavone was recently isolated from the methanol leaf extract of *L. sericea* with significant broad-spectrum antibacterial activity at 1.95–125 μg mL^–1^ (Pendota et al., 2018). This study investigated the effect of seasonal changes on the anti-acne activity of the leaf extracts using untargeted NMR-based metabolomics and subsequent LC-MS analysis. The winter samples provided the best anti-acne activity with a mean value of 5.20 μg mL^–1^, comparable to tetracycline (positive drug control) at 3.90 μg mL^–1^, also comparing well to previous reports (Sharma et al., 2014). This activity is, however, much better than anti-acne activity found in herbs such as rose (*Rosa damascene*), dzong (*Eucommia ulmoides* oliv.) and yerba mate (*Ilex paraguariensis*) with MIC values of 1 to 2.5 μg mL^–1^ (Chomnawang et al., 2007; Kim et al., 2008b; Tsai et al., 2010). The winter samples, however, presented better activity than the summer and spring extracts with much lower mean MIC values of 26.04 μg mL^–1^ and 20.83 μg mL^–1^, respectively (Table 1). The activity of the summer extract corroborates with previous reports where *L. sericea* leaves inhibited *P. acnes* with MIC values of ≤15.62 μg mL^–1^. The autumn and winter extracts (7.81 and 5.20 μg mL^–1^, respectively) therefore both showed increased activity to the previous reports, although not significantly different, and is an indication of the preparation of the plant for the colder months as better anti-bacterial activity has been obtained already in autumn. As the seasons progressed from spring to winter, the activity in winter and autumn improved significantly when compared to spring and summer samples. The improved activity from the warmer to the colder months could not be related to the previously identified anti-acne compounds, but to flavonoid-type compounds not detected in the spring and summer months. The effect of season has been investigated by numerous authors, as each plant reacts differently to seasonal changes and different metabolic pathways are responsible for production of the various secondary metabolites in both colder and warmer months (Prinsloo and Nogemane, 2018). Plants such as *Rosmarinus officinalis*, *Salvia fruticose*, and *Croton heliotropiifolius* contain higher concentrations of active compounds in summer and spring months (Lemos et al., 2015; Sarrou et al., 2016; de Alencar Filho et al., 2017). On the contrary many reports described increased activity and active compound levels in the colder months for example *Calamintha nepeta*, *Phillyrea angustifolia*, and *Thymus longicaulis* (Scognamiglio et al., 2014; Pacifico et al., 2015), whereas in some plants seasonal variation does not affect the chemical composition such as in *Vaccinium myrtillus* (Bujor et al., 2016). In this study, improved activity in colder months is an indication that the plant is preparing for colder seasons by increased production of anti-bacterial compounds, and therefore better activity was achieved in autumn and winter, even though activity in winter was superior at 5.20 μg mL^–1^. This study is the first report where the effect of season was determined on inhibitory activity of *L. sericea* against *P. acnes*.

The PCA provided an unsupervised overview of the metabolome of the plant leaves harvested in spring, autumn, winter and summer seasons, where variation was observed and samples similar in chemical profiles grouped together. The winter and autumn samples were clustered close together even though it was separated into different groups (Figure 1). The main compounds annotated in the extracts were alanine, acetamide, choline, glycine, fumarate, isoleucine, sucrose, and xylose, based on annotation using the Chenomx database and previously published data (Table 2). The tight clustering of the winter and autumn samples indicated low variation among these samples, whereas the loose grouping of the spring and summer samples indicated much more variation. Informed by the anti-acne results showing that the winter extracts have the best anti-bacterial activity at the MIC value of 5.20 μg mL^–1^ and spring with the lowest activity at 26.04 μg mL^–1^ (Table 1), these seasons were selected for a supervised OPLS-DA analysis to determine the difference in the chemical composition of the samples harvested in the different seasons (Figure 2). The OPLS-DA score plots clearly separated the spring from the winter samples, with a very good model prediction ability (*R*^2^X = 0.914 and *R*^2^Y = 0.897). Validation of the model was determined with the permutation test, performed with 100 permutations and indicated that the model is valid (Figure 3). Due to the clear separation of the samples, it was possible to construct a contribution plot of the winter samples (Figure 4), showing important contributing regions responsible for clustering of the samples. The aromatic (δ 6.1–8.0), sugar (3.3–4.2) and aliphatic regions (δ 1.2–1.3) were strongly associated with the winter samples. The peaks in these regions were compared with NMR profiles in Chenomx and Human Metabolome Database and also with previously published data. It was concluded that the regions corresponds very well to a flavonoid-type compound, which was confirmed with the use of LC-MS analysis. LC-MS analysis was imported to MAGMa, to determine the best possible fit to a structure which could be differentiated between the winter and spring samples. MAGMa is a highly acclaimed computational method annotating all fragmented compounds in LC-MSn data sets with candidate molecules taken from large chemical databases such as PubChem or the Human Metabolite Database. Based on an algorithm for candidate substructure annotation of multistage accurate mass spectral trees, provides candidates ranked on the calculated matching score (Ridder et al., 2013, 2014). The polymethoxylated flavone 2-(4-ethoxyphenyl)-5,6,7,8-tetramethoxy-4H-1-benzopyran-4-one (Figure 5) achieved the highest matching score. The fragmentation tree of this compound was then transferred to CSIFingerID, which supported the proposed compound in MAGMa. Furthermore, the LC-MS analysis also showed difference in comparison of the height of the peak M/z 386.137 of the winter and spring samples, confirming a difference in compound concentration, which was indeed in a higher concentration in the winter samples when compared to the spring samples. The compound, however, was not isolated from the extract and the proposed compound should be isolated and subsequently confirmed with 2-D NMR and LC-MS analysis.

**FIGURE 6:**
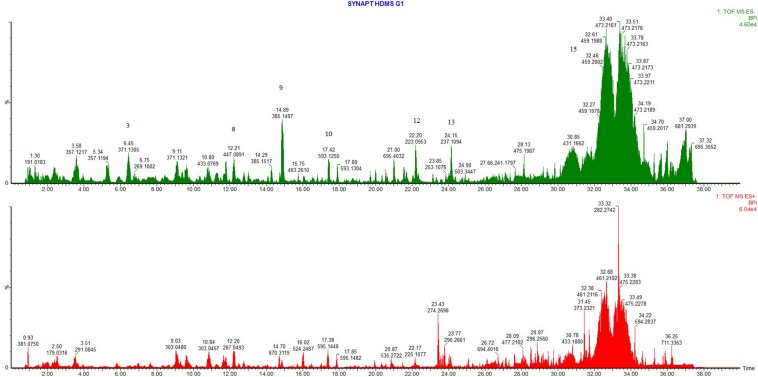
Ultra-high-performance liquid chromatography coupled with mass spectrometry (UHPLC-MS) base peak intensity/ion (BPI) chromatograms [electrospray ionization, ESI (–) in green and ESI (+) in red]. The compounds are indicated on the figure for tangeritin, (3), kaempferol-7-O-β-D-glucopyranoside (8) 2-(4-ethoxyphenyl)-5,6,7,8-tetramethoxy-4H-1-benzopyran-4-one (9), tiliroside (10), aspidinol B (aspidinol) (12), aspidinol C or aspidinol D (13), and α-kosin (15).

**FIGURE 7:**
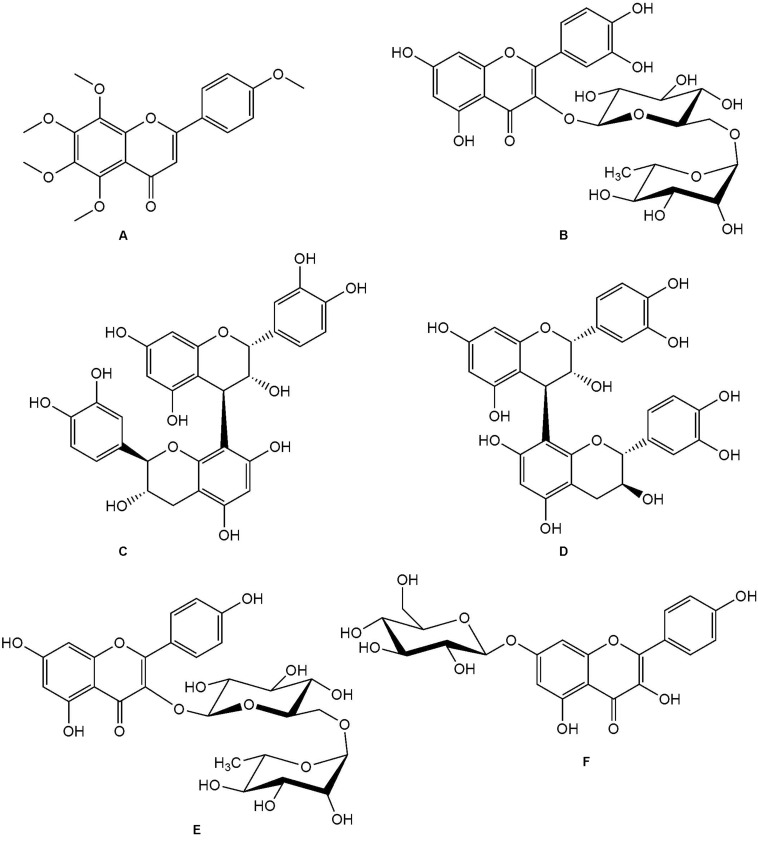
Structures of compounds not previously isolated from *L. seriea*. **(A)** tangeretin; **(B)** rutin; **(C)** procyanidin B1; **(D)** procyanidin B2; **(E)** kaempferol-3-O-rutinoside (nicotiflorin); **(F)** kaempferol-7-O-β-D-glucopyranoside.

The extracted ion chromatograms (XICs) obtained based on the newly proposed unknown compound and known compounds was found in published literature. The accurate mass data, elemental composition, DBE as well as isotopic fit calculations matched the compounds as presented in Table 4. Fragmentation spectra were also submitted to the NIST mass spectral library (Stein, 2014, Version 2.2 build Jun 2014) as well as the mass spectral libraries developed on the Synapt G1 system for identification of the compounds (Figure 6). The list of compounds includes compounds that were previously identified from the plant such as α-kosin (15) (Sharma et al., 2014), 3,5,7,3′,4′-pentahydroxyflavone, tiliroside (10), 1-hydroxy-2-oxopomolic acid, 5,7-dihydroxychromone (Pendota et al., 2018), aspidinol (12), and desaspidinol (Bosman et al., 2004). Compounds not previously identified from *L. sericea* includes-(4-ethoxyphenyl)-5,6,7,8-tetramethoxy-4H-1-benzopyran-4-one (9), tangeritin (3), rutin, quercetin glucoside and kaempferol glucosides (8) (Figure 7). Phloroglucinol derivatives aspidinol and aspidin BB have also shown antibacterial (Pendota et al., 2018) as well as potent anti-acne activity (Gao et al., 2016).

Information on the compound 2-(4-ethoxyphenyl)-5,6,7,8-tetramethoxy-4H-1-benzopyran-4-one (both on structure elucidation and biological activity) is very limited and therefore information on the possible biological activity of the compound was derived from similar polymethoxylated flavones found in citrus, supported by the isolation of 3,5,7,3′,4′-pentahydroxyflavone from *L. sericea* (Pendota et al., 2018). Tangeretin and 2-(4-ethoxyphenyl)-5,6,7,8-tetramethoxy-4H-1-benzopyran-4-one are very similar compounds with only a difference of the ethoxy group substituted with a methoxy group in tangeretin. Hesperidin, nobiletin and derivatives are also very similar compounds found in citrus fruit and peel in tangerine, lemon and orange. Hesperidin was found to be the most predominant flavonoid in tangerine peel, followed by tangeretin and nobiletin (Ho and Kuo, 2014). Even though pharmacological activity such as neuroinflammatory activity, antibacterial, antifungal, antiaging, anti-tyrosinase, anti-acne, antiallergic, anticancer, anti-inflammatory and antiviral activity against the human respiratory syncytial virus (RSV) have been assigned to these compounds, it is known that these compounds individually often do not account for the activity of the extracts, and that these compounds in combination is responsible for the pharmacological activities observed (Kim et al., 2008a; Ho and Kuo, 2014; Rashed et al., 2015; Xu et al., 2015; Vargas et al., 2016; Adhikari et al., 2017; Hagenlocher et al., 2017). Additionally tangeretin and nobiletin also showed the best antibacterial activity against *Staphylococcus aureus*, *Microsporum canis*, *Escherichia coli*, *Trichophyton mentagrophytes* when tested against bacterial and fungal species when the peel extracts of *C. sinensis*, *C. limon*, and *C. reticulata* were tested, with antimicrobial activity also reported for these compounds in the leaf extract of *C. volkameriana* (Johann et al., 2007; Rashed et al., 2015). These two compounds are also the most effective compounds at inhibiting cancer cell growth of melanoma and lung (Rashed et al., 2015).

The essential oils of *C. obovoides* and *C. natsudaidai* were evaluated for their possibility as skin treatment when tested against *P. acnes* and *S. epidermidis*, exhibiting very good antibacterial activity against both bacteria with MIC values of 0.31 μL mL^–1^ and 2.5–10 μg mL^–1^, respectively (Kim et al., 2008a). The anti-acne activity of *Citrus* species was further confirmed with essential oils of *C. limon* and *C. paradisi*, showing anti-acne properties against *P. acnes* (Zu et al., 2010) while essential oil of *C. aurantium* is known for its use in the treatment of acne (Sinha et al., 2014). *Citrus junos* was also investigated for its use in the cosmeceutical industry and the active compounds such as tangeretin and hesperidin were found to have anti-aging and anti-tyrosinase activity (Adhikari et al., 2017). Literature therefore provides ample evidence of the anti-bacterial activity of the polymethoxylated flavones, and although the activity of 2-(4-ethoxyphenyl)-5,6,7,8-tetramethoxy-4H-1-benzopyran-4-one has not been determined previously, the compound could possess very similar activity to the citrus polymethoxylated flavones such as tangeretin and nobiletin although it should be confirmed in biological assays.

## Conclusion

In conclusion, the leaf extracts of *L. sericea* inhibited the growth of *P. acnes* with a significant mean MIC value of 5.20 μg mL^–1^ (comparable to the positive control tetracycline) for winter with lower activity during autumn, spring and summer. This report confirms the effect of external environmental stimuli on the chemical profile and associated biological activity of plants, and in particular *L. sericea*. Untargeted NMR-based metabolomics was applied to successfully detect differences in the metabolome of the plant samples collected in different seasons. Multivariate data analysis (PCA and OPLS-DA) indicated a clear separation of samples according to seasons, corroborating the results obtained in the anti-acne assay. Subsequent analysis of the contribution plot, identified NMR regions in the aromatic, sugar and aliphatic regions, corresponding to a flavonoid-type structure. Following this, LC-MS, MAGMa and CSIFingerID were further used to propose the compound 2-(4-ethoxyphenyl)-5,6,7,8-tetramethoxy-4H-1-benzopyran-4-one as the compound responsible for the separation of the samples in the metabolomic analysis and better activity in winter. This compound is not very common and not well-researched, however, evidence of anti-bacterial activity is available for similar compounds such as tangeretin also indicating the possible anti-bacterial activity as was observed in the bacterial assay. UHPLC linked to LC-MS analysis was used to confirm the presence of 2-(4-ethoxyphenyl)-5,6,7,8-tetramethoxy-4H-1-benzopyran-4-one, tangeretin as well as compounds previously isolated from *L. sericea* such as α-kosin, 3,5,7,3′,4′-pentahydroxyflavone, tiliroside, and aspidinol. Future work should focus on isolation of the compound to confirm the structure of this relatively unknown compound 2-(4-ethoxyphenyl)-5,6,7,8-tetramethoxy-4H-1-benzopyran-4-one, and should further investigate the anti-acne activity of the compound and its possible synergistic effects with compounds such as kojic acid, tangeretin, aspidinol and 3,5,7,3′,4′-pentahydroxyflavone, already identified from this plant which exhibited strong anti-bacterial activity.

## Data Availability Statement

The dataset “*Leucosidea sericea* NMR seasons” for the metabolomic analysis of this study can be found in the Mendeley Library http://dx.doi.org/10.17632/pyfh5ykgrw.1.

## Author Contributions

PS conducted the research, analyzed and interpreted the data, and drafted the initial manuscript. NL provided assistance in conceptualization of the project and the anti-acne assays. GP conceptualized the research, assisted with the metabolomic analysis and interpretation of results, and finalized the manuscript for submission.

## Conflict of Interest

The authors declare that the research was conducted in the absence of any commercial or financial relationships that could be construed as a potential conflict of interest.

## Footnotes

1 www.hmdb.ca/

2 http://www.emetabolomics.org/magma

3 http://www.csi-fingerid.org/

4 https://www.nmrdb.org

## References

[B1] Adhikari D. Panthi V. K. Pangeni R. Kim H. J. Park J. W. (2017). Preparation, characterization, and biological activities of topical anti-aging ingredients in a *Citrus junos* callus extract. *Molecules* 22:2198. 10.3390/molecules22122198 29232889 PMC6149992

[B2] Aremu A. O. Fawole O. A. Chukwujekwu J. C. Light M. E. Finnie J. F. Van Staden J. (2010). In vitro antimicrobial, anthelmintic and cyclooxygenase-inhibitory activities and phytochemical analysis of *Leucosidea sericea*. *J. Ethnopharmacol.* 131 22–27. 10.1016/j.jep.2010.05.043 20542105

[B3] Banfi D. Patiny L. (2008). Resurrecting and processing NMR spectra on-line. *Chimia* 62 280–281. 10.2533/chimia.2008.280

[B4] Bosman A. A. Combrinck S. Roux-Van Der Merwe R. Botha B. M. McCrindle R. I. (2004). Isolation of an anthelmintic compound from *Leucosidea sericea*. *South Afr. J. Bot.* 70 509–511. 10.1016/S0254-6299(15)30189-7

[B5] Bujor O. Le C. Volf I. Popa V. I. Dufour C. (2016). Seasonal variations of the phenolic constituents in bilberry (*Vaccinium myrtillus* L.) leaves, stems and fruits, and their antioxidant activity. *Food Chem.* 213 58–68. 10.1016/j.foodchem.2016.06.042 27451155

[B6] Chomnawang M. T. Surassmo S. Nukoolkarn V. S. Gritsanapan W. (2007). Effect of Garcinia mangostana on inflammation caused by *Propionibacterium acnes*. *Fitoterapia* 78 401–408. 10.1016/j.fitote.2007.02.019 17644272

[B7] de Alencar Filho J. M. T. da C. Araújo L. Oliveira A. P. Guimarães A. L. Pacheco A. G. M. (2017). Chemical composition and antibacterial activity of essential oil from leaves of *Croton heliotropiifolius* in different seasons of the year. *Rev. Bras. Farmacogn.* 27 440–444. 10.1016/j.bjp.2017.02.004

[B8] Eloff J. N. (1998). A sensitive and quick microplate method to determine the minimal inhibitory concentration of plant extracts for bacteria. *Planta Med.* 64 711–713. 10.1055/s-2006-957563 9933989

[B9] Gao C. Guo N. Li N. Peng X. Wang P. Wang W. (2016). Investigation of antibacterial activity of aspidin BB against Propionibacterium acnes. *Arch. Dermatol. Res.* 308 79–86. 10.1007/s00403-015-1603-x 26596576

[B10] Hagenlocher Y. Feilhauer K. Schäffer M. Bischoff S. C. Lorentz A. (2017). Citrus peel polymethoxyflavones nobiletin and tangeretin suppress LPS- and IgE-mediated activation of human intestinal mast cells. *Eur. J. Nutr.* 56 1609–1620. 10.1007/s00394-016-1207-z 27021766

[B11] Hardy N. W. Hall R. D. (2012). *Plant Metabolomics: Methods and Protocols.* Totowa, NJ: Humana Press.

[B12] Ho S. C. Kuo C. T. (2014). Hesperidin, nobiletin, and tangeretin are collectively responsible for the anti-neuroinflammatory capacity of tangerine peel (*Citri reticulatae pericarpium*). *Food Chem. Toxicol.* 71 176–182. 10.1016/j.fct.2014.06.014 24955543

[B13] Johann S. De Oliveira V. L. Pizzolatti M. G. Schripsema J. Braz-Filho R. Branco A. (2007). Antimicrobial activity of wax and hexane extracts from *Citrus* spp. peels. *Mem. Inst. Oswaldo Cruz* 102 681–685. 10.1590/S0074-02762007000600004 17923995

[B14] Kim H. K. Choi Y. H. Verpoorte R. (2010). NMR-based metabolomic analysis of plants. *Nat. Protoc.* 5 536–549. 10.1038/nprot.2009.237 20203669

[B15] Kim S.-S. Baik J. S. Oh T.-H. Yoon W.-J. Lee N. H. Hyun C.-G. (2008a). Biological activities of Korean *Citrus obovoides* and *Citrus natsudaidai* essential oils against acne-inducing bacteria. *Biosci. Biotechnol. Biochem.* 72 2507–2513. 10.1271/bbb.70388 18838824

[B16] Kim S.-S. Kim J.-Y. Lee N. H. Hyun C.-G. (2008b). Antibacterial and anti-inflammatory effects of Jeju medicinal plants against acne-inducing bacteria. *J. Gen. Appl. Microbiol.* 54 101–106. 10.2323/jgam.54.101 18497484

[B17] Lemos M. F. Lemos M. F. Pacheco H. P. Endringer D. C. Scherer R. (2015). Seasonality modifies rosemary’ s composition and biological activity. *Ind. Crop. Prod.* 70 41–47. 10.1016/j.indcrop.2015.02.062

[B18] Maree J. E. Viljoen A. M. (2012). Phytochemical distinction between *Pelargonium sidoides* and *Pelargonium reniforme* — A quality control perspective. *South Afr. J. Bot.* 82 83–91. 10.1016/j.sajb.2012.07.007

[B19] Mediani A. Abas F. Khatib A. Maulidiani H. Shaari K. Choi Y. H. (2012). 1H-NMR-based metabolomics approach to understanding the drying effects on the phytochemicals in *Cosmos caudatus*. *Food Res. Int.* 49 763–770. 10.1016/j.foodres.2012.09.022

[B20] Moco S. Vervoort J. Moco S. Bino R. J. De Vos R. C. H. Bino R. (2007). Metabolomics technologies and metabolite identification. *TrAC Trends Anal. Chem.* 26 855–866. 10.1016/j.trac.2007.08.003

[B21] Pacifico S. Galasso S. Piccolella S. Kretschmer N. Pan S. Marciano S. (2015). Seasonal variation in phenolic composition and antioxidant and anti-inflammatory activities of *Calamintha nepeta* (L .) Savi. *Food Res. Int.* 69 121–132. 10.1016/j.foodres.2014.12.019

[B22] Pendota S. C. Aremu A. O. Po L. Rárová L. Dole K. Van Staden J. (2018). Identification and characterization of potential bioactive compounds from the leaves of *Leucosidea sericea*. *J. Ethnopharmacol.* 220 169–176. 10.1016/j.jep.2018.03.035 29604376

[B23] Prinsloo G. Nogemane N. (2018). The effects of season and water availability on chemical composition, secondary metabolites and biological activity in plants. *Phytochem. Rev.* 17 889–902. 10.1007/s11101-018-9567-z

[B24] Rashed K. Said A. Zheng Y.-T. Tawila A. Fouche G. El-fiky N. M. (2015). Anticancer, Anti HIV-1 and antimicrobial potentials of methanol extract and non polar fractions of *Citrus volkameriana* leaves and phytochemical composition. *Res. J. Med. Plant* 9 201–214. 10.3923/rjmp.2015.201.214

[B25] Ridder L. Van Der Hooft J. J. J. Verhoeven S. De Vos R. C. H. Bino R. J. Vervoort J. (2013). Automatic chemical structure annotation of an LC-MSn based metabolic profile from green tea. *Anal. Chem.* 85 6033–6040. 10.1021/ac400861a 23662787

[B26] Ridder L. Van Der Hooft J. J. J. Verhoeven S. De Vos R. C. H. Vervoort J. Bino R. J. (2014). In silico prediction and automatic LC-MSn annotation of green tea metabolites in urine. *Anal. Chem.* 86 4767–4774. 10.1021/ac403875b 24779709

[B27] Sarrou E. Martens S. Chatzopoulou P. (2016). Metabolite profiling and antioxidative activity of Sage (*Salvia fruticosa* Mill.) under the influence of genotype and harvesting period. *Ind. Crop. Prod.* 94 240–250. 10.1016/j.indcrop.2016.08.022

[B28] Scognamiglio M. D’Abrosca B. Fiumano V. Golino M. Esposito A. Fiorentino A. (2014). Seasonal phytochemical changes in *Phillyrea angustifolia* L.: metabolomic analysis and phytotoxicity assessment. *Phytochem. Lett.* 8 163–170. 10.1016/j.phytol.2013.08.012

[B29] Sharma R. Kishore N. Hussein A. Lall N. (2014). The potential of *Leucosidea sericea* against *Propionibacterium acnes*. *Phytochem. Lett.* 7 124–129. 10.1016/j.phytol.2013.11.005

[B30] Sinha P. Srivastava S. Mishra N. Yadav N. P. (2014). New perspectives on antiacne plant drugs: contribution to modern therapeutics. *Biomed Res. Int.* 2014:301304. 10.1155/2014/301304 25147793 PMC4132408

[B31] Stein S. E. (2014). *Mass Spectral Database and Software Version 2.2. National Institute of Standards and Technology (NIST)*. Gaithersburg, MD: Scientific Instrument Services.

[B32] Tsai T. H. Tsai T. H. Wu W. H. Tseng J. T. P. Tsai P. J. (2010). In vitro antimicrobial and anti-inflammatory effects of herbs against *Propionibacterium acnes*. *Food Chem.* 119 964–968. 10.1016/j.foodchem.2009.07.062

[B33] Vargas I. SAanz I. Moya P. Prima-Yufera E. (2016). Antimicrobial and antioxidant compounds in the nonvolatile fraction of expressed orange essential oil. *J. Food Prot.* 62 929–932. 10.4315/0362-028x-62.8.929 10456748

[B34] Xu J. J. Liu Z. Tang W. Wang G. C. Chung H. Y. Liu Q. Y. (2015). Tangeretin from citrus reticulate inhibits respiratory syncytial virus replication and associated inflammation in vivo. *J. Agric. Food Chem.* 63 9520–9527. 10.1021/acs.jafc.5b03482 26468759

[B35] Zu Y. Yu H. Liang L. Fu Y. Efferth T. Liu X. (2010). Activities of ten essential oils towards *Propionibacterium acnes* and PC-3, A-549 and MCF-7 cancer cells. *Molecules* 15 3200–3210. 10.3390/molecules15053200 20657472 PMC6263286

